# Particle-based model shows complex rearrangement of tissue mechanical properties are needed for roots to grow in hard soil

**DOI:** 10.1371/journal.pcbi.1010916

**Published:** 2023-03-07

**Authors:** Matthias Mimault, Mariya Ptashnyk, Lionel X. Dupuy

**Affiliations:** 1 Information and Computational Science, The James Hutton Institute, Invergowrie, United Kingdom; 2 School of Mathematical and Computer Sciences, Maxwell Institute for Mathematical Sciences, Heriot-Watt University, Edinburgh, United Kingdom; 3 Neiker, Basque Institute for Agricultural Research and Development, Derio, Spain; 4 Ikerbasque, Basque Foundation for Science, Bilbao, Spain; Leiden University Faculty of Science: Universiteit Leiden Faculteit der Wiskunde en Natuurwetenschappen, NETHERLANDS

## Abstract

When exposed to increased mechanical resistance from the soil, plant roots display non-linear growth responses that cannot be solely explained by mechanical principles. Here, we aim to investigate how changes in tissue mechanical properties are biologically regulated in response to soil strength. A particle-based model was developed to solve root-soil mechanical interactions at the cellular scale, and a detailed numerical study explored factors that affect root responses to soil resistance. Results showed how softening of root tissues at the tip may contribute to root responses to soil impedance, a mechanism likely linked to soil cavity expansion. The model also predicted the shortening and decreased anisotropy of the zone where growth occurs, which may improve the mechanical stability of the root against axial forces. The study demonstrates the potential of advanced modeling tools to help identify traits that confer plant resistance to abiotic stress.

## Author summary

The lack of suitable approaches for studying how plant roots adapt to mechanical resistance from soils is limiting our ability to adapt cropping system to climate change. Drought resistance for example is enhanced both by deep rooting and the ability to grow through dry layers of soil. In this study, we overcome experimental limitations to measure tissue mechanics *in-situ* with a computational model able to solve simultaneously the physical process of growth macroscopically and the behavior of individual cells in the meristem. The study reveals that complex rearrangement of tissues mechanical properties may occurs in response to increased mechanical resistance from the soil. Results show the potential of particle-based models to compute the growth of an entire organ at cellular resolution and that such models could significantly advance the studies of plant morphogenesis.

## 1 Introduction

**Fig 1:**
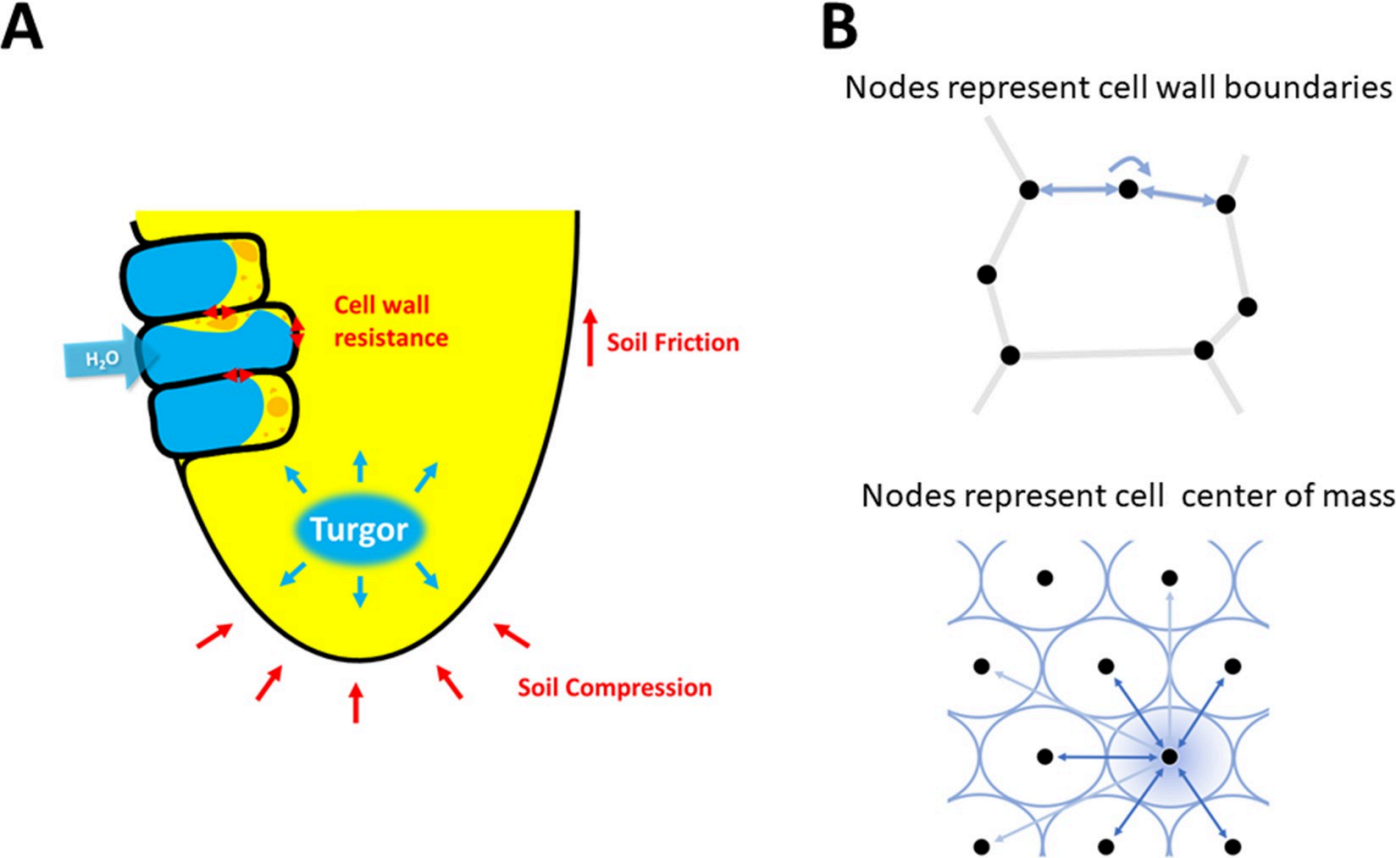
Biophysics of root growth in soil. (A) Roots grow when turgor pressure exceeds the mechanical resistance of cell walls and the friction and compression from the soil. (B) In cellular models of plant tissues (top), cells are divided into cell wall segments and the balance of forces is computed on each vertex [9]. A simplifying approach is to represent the cell as a point in space (particle) and to define the interactions between other cells as a function of the size of the cell, its distance to neighbors and its shape (bottom). Mathematically, a kernel function is defined to interpolate field variables such as mechanical stress (turgor pressure or tensile stress in cell walls) or velocity, and numerical approximation of particle dynamics reduces to the weighted sum of variables attached to each particle, with the weight given by a kernel function.

Root growth results from two opposing biophysical forces (Fig 1). On the one hand, gradients in osmotic potential drive water into the cytoplasm and cause a build-up of turgor pressure [1]. On the other hand, tension in cell walls [2], friction forces at root surfaces [3] and compression from the soil [4] oppose the turgor pressure and determine how the tissue grows. Turgor pressure is also affected by factors such as soil matric potential and the hydraulic conductivity of the tissue [5]. The biophysical mechanisms of root-soil interaction are now well formalized. Turgor pressure in cells and tissues increases as a result of water and ion transport through cell membranes [6], whilst the stretching of the cell wall is defined classically using viscoplastic models [7]. The soil water content is described by soil mechanical and hydraulic models such as Mohr Coulomb and Richard’s equations [8].

Yet, root responses to soil mechanical resistance are not well explained by models. Experiments consistently record maximum pressures exerted by a growing root at approximately 1 MPa [10], but roots are observed growing in soils whose mechanical resistance is measured well over 5 MPa [11]. Models developed from first principles [12] concluded that an osmoregulation against the soil resistance or the matric potential was needed to predict adequate root growth rate but, since then experiments by [13,14,15] showed turgor pressure increased by no more than 0.2 MPa when roots are arrested or strongly impeded. It has been suggested that gaps between turgor and soil pressures are due to an overestimation of the friction coefficient of root-soil interfaces from penetrometer resistance tests [3]. However, roots exhibit non-linear growth responses to soil resistance [15,16] that are incompatible with linear responses expected from a friction process.

Current limitations in modeling are due to a lack of understanding on how developmental or morphological responses link to adaptation to mechanical impedance from the soil. In hard soils, the growth zone is reduced in size [15]. The root diameter increases significantly with soil strength, supposedly to reduce axial mechanical stress and prevent buckling [17]. The shape of root tips has also been associated with the ability to overcome mechanical resistance of soils [4]. But to date, it has not been possible to model interactions between root traits and increased mechanical resistance from the soil. Soils are granular media and particles exert forces that are discrete and heterogeneous. They create opportunities for plant roots to exploit paths of least resistance [1] and it is difficult to quantify how roots can exploit them to overcome macroscopic pressure from the soil.

A main challenge is to develop models able to grasp the complex relations between root traits and soil properties. A suitable model must describe interactions at the microscopic scale (cells, soil particles) but resolve emerging processes at the macroscopic scale, across entire root system and under changing soil conditions [18]. In this study we have developed a simple and efficient computational framework based on the Smooth Particle Hydrodynamics method (SPH) to model the root response to mechanical resistance of the soil. The model associates the SPH particle to a single cell and can compute entire root meristems. The study explores the relationships between soil strength and the softening of the tissue as one of the plausible mechanisms involved in root responses to mechanical forces [2]. Simulations then identify the modification of mechanical properties required to explain observed root elongation rates.

## 2 Results

### 2.1 SPH computations enable modelling of entire root meristem at cellular resolution

**Fig 2:**
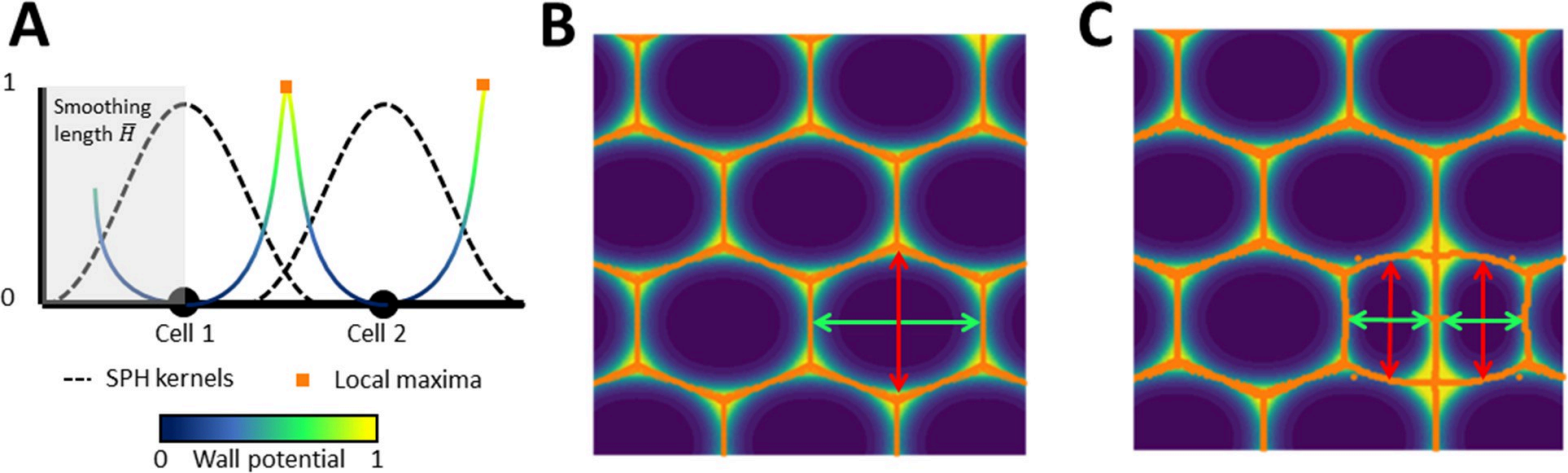
The particle model. (A) The interaction between cells is described through the kernel function (blue) associated with the particle (see Method A in S1 Text), which is a function that decreases with the distance from the particle center with the smoothing length. $\bar{H}$. It can be used to recover various field quantities, here the wall potential which indicates the likeliness of the position of the cell wall. (B) Ellipsoids define the size and shape of the cell. An ellipsoid is defined by its principal axes (here long in green, short in red) and used to generate the wall potential (background color, yellow being the most probable location of a wall and orange being the ridge). (C) The cell division occurs along the longest axis and split the mother cell into equally sized daughter cells.

The SPH model derived here represents each cell *i* within the root tip as a particle with center of mass *x*_*i*_ and with cell shape represented as an ellipsoid with the shape matrix *Q*_*i*_, see Method A in S1 Text for a full description. The particles do not bear topological relation to one another, but state variables such as the velocity field and tissue density across the root continuum can be computed from kernel-based interpolation between particles which divide as a result of tissue growth (Fig 2). The interpolation relies on a kernel function, a function decreasing with the distance from the center of the particle, to weight the effect of a particle onto its neighbors and compute the value of the state variables [19]. Kernel-based interpolation is used in the SPH method to integrate constitutive equations from the continuum mechanics and to determine an approximation of the evolution of state variables (see Method A in S1 Text).

The model we propose represents growth as a source of mass in the conservation equation (Eq 1). The source of mass is a function of the stretching of the tissue and is controlled by the softening coefficient λ_i_ defined at each cell *i*. To maintain the cellular architecture of the tissue during growth, the shape of a cell is represented as an ellipsoid matrix *Q*_*i*_ (Fig 2B and 2C). The model triggers a cell division when the principal axis characterized by the matrix *Q*_*i*_ reaches a division threshold $\bar{l_{I}}$, with the division oriented perpendicularly to it. An anisotropic definition of mechanical properties of the tissue, defined through the compliance tensor *S*_*i*_, enables the model to represent a broad range of morphogenetic processes.

To keep computations tractable, the representation of the root was simplified in various ways. First, we did not explicitly represent the soil as a solid structure interacting with the cells and growing tissues. Instead, we applied a uniform pressure on the tissue, termed pressure differential *p*, which represents the difference between turgor pressure in plant cells, and the perceived resistance from the soil. To facilitate the parameterization of the model, we also chose to focus on the process of root axial and radial growth and ignored several anatomical features important to root functions, including the root cap or the formation of specialized cells such as root hairs. We also assumed root properties vary only as a function of the distance along the root and the pressure differential.

With these assumptions, the model could be easily parameterized using experimental data available from the literature. One main difficulty of this task was to establish how coefficients in constitutive equations, e.g., λ_I_, *Q*_*i*_ and *l*_*i*_, vary along the root and as a function of external forces. Because of the computational complexity of the model, the approach employed was to determine spatial variation in each coefficient using bell-shaped or sigmoid functions that are estimated independently from each other. Coarse estimates of parameters in these functions were determined by trial and error, before a final adjustment was made using observed relationships between the model parameters and features of the growth curves obtained experimentally (e.g., inflection points, asymptotic values, size of transition).

### 2.2 Coordination of radial and axial growth is needed to maintain the shape of the tip of growing tissues

**Fig 3:**
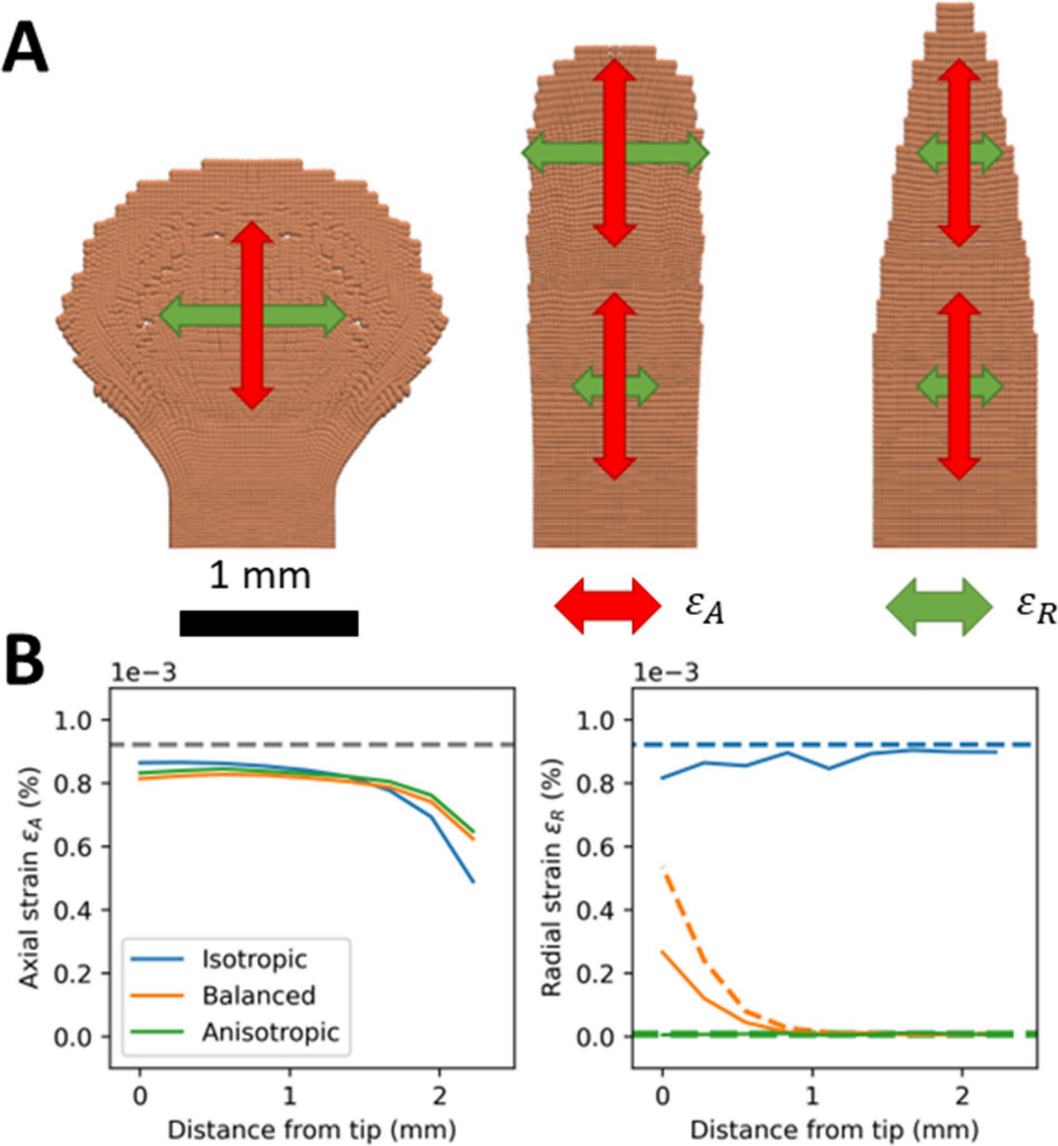
Impact of anisotropy of mechanical properties on the morphology of a growing tissue. (A) Three cases were used to test the ability of the model to describe tissue morphogenesis. The isotropic case (A, left) was modeled using a radial Young modulus *E*_*R*_ equal to the axial Young modulus *E*_*A*_. The anisotropic case (A, right) was modeled using *E*_*R*_ equal to the maximal radial Young modulus *E*_*H*_. The balanced case (A, center) was modeled using a *E*_*R*_ increasing smoothly from *E*_*A*_ at the tip to *E*_*H*_ at the base, using parameters shown in Table *1*. Arrows describe the resulting strain rates in the growing tissue with the axial strain *ε*_*A*_ shown in red and radial strain *ε*_*R*_ shown in green. Simulations were initiated with particles distributed on a 2D cartesian lattice. (B) Comparison of SPH predictions with analytical solutions for the isotropic, anisotropic, and balanced cases for axial strain (B, left) and radial strain (B, right). Dashed curves represent analytical solutions and solid curve numerical results. The isotropic, balanced, and anisotropic cases are represented in blue, green, and orange, respectively. Analytical solutions are computed from Eq 8.

To understand the factors that determine the maintenance of root tip properties through time, we first examined how tissue anisotropy links to root morphology. Simulations examined how the radial Young modulus *E*_*R*_ in Eq 4 affects the shape of a growing tissue. Results showed (Fig 3) that when *E*_*R*_ was equal to the axial Young modulus *E*_*A*_, isotropic expansion was observed at the tip, which results in a spherical outgrowth (Fig 3A, left). On the other hand, when *E*_*R*_ was significantly larger than *E*_*A*_, uni-axial expansion was observed, transforming the parabolic shape of the tissue into an increasingly sharp structure (Fig 3A, right). Therefore, we concluded that parameters that maintain stable diameter and tip shape for at least 8 hours represent biologically acceptable description of the root. In our simulations we found sigmoid-shaped functions adequately describe the transition from isotropic growth at the tip to anisotropic growth in the root elongation zone.

We use a sigmoid function to describe how the radial modulus *E*_*R*_ vary along the root (Eq 6 and Figure A in S1 text), and these variations enabled the model to produce a root tip with parabolic shape, specifying the transition from the cell division zone to the root elongation zone (Fig 3A, center). The inflection point of the sigmoid curve *x*_*e*_ was found to be 0.6 mm so that the transition occurred within half a mm from the growing tip. The slope *s*_*e*_ was found to be 5 and the baseline constant *c*_*e*_ was 0.

The accuracy of the numerical solutions was tested against three study cases, an isotropic, an anisotropic, and a configuration where mechanical properties vary along the tissue (balanced Fig 3B). In all cases, the axial strain computed analytically was constant along the tissue. The SPH model predicted well the axial component of the strain close to the tip (RE~10%, *x*<1.5mm). The accuracy of predictions for the axial strain reduced slightly at *x* = 2 mm, which is the location of boundary particles. Since particles are fixed at the boundary, the movement of the neighboring particles was constrained. The SPH model predicted well the radial strain in the isotropic case (RE~5%). The accuracy of the prediction in the balanced case was reduced due to the smaller number of particles at the tip (RE~33%). The accuracy in the anisotropic case was lower because growth was small, and the model was more sensitive to numerical errors (RE~24%).

### 2.3 Root development in unimpeded conditions

We identified parameters that fitted experimental data from *Arabidopsis thaliana* roots, 6 days after planting, using axial strain rate data, cell length data and cell division rate data derived from [20]. The fitting of the model was achieved following the method detailed in section 4.5 of the material and methods where the softening coefficient *λ* is adjusted first to fit the strain rate data, the anisotropy of the tissue *E*_*R*_ is then fitted to root diameter data and the critical cell length $\bar{l}$ is adjusted to the cell division rate. The cell division was fitted only on the first 500 μm from the root tip. At distance larger than 500 μm, the cell division threshold $\bar{l}$ was kept constant to avoid excessively large cells which were more prone to instability. Consequently, the model overestimated the cell division rate in the more distal regions of the root. Another observation was that cell division in the outer layer of the tissue occurred earlier. This was due to the radial expansion of the tissue, which stretch more in the cells in outer layers than in the inner layers.

**Fig 4:**
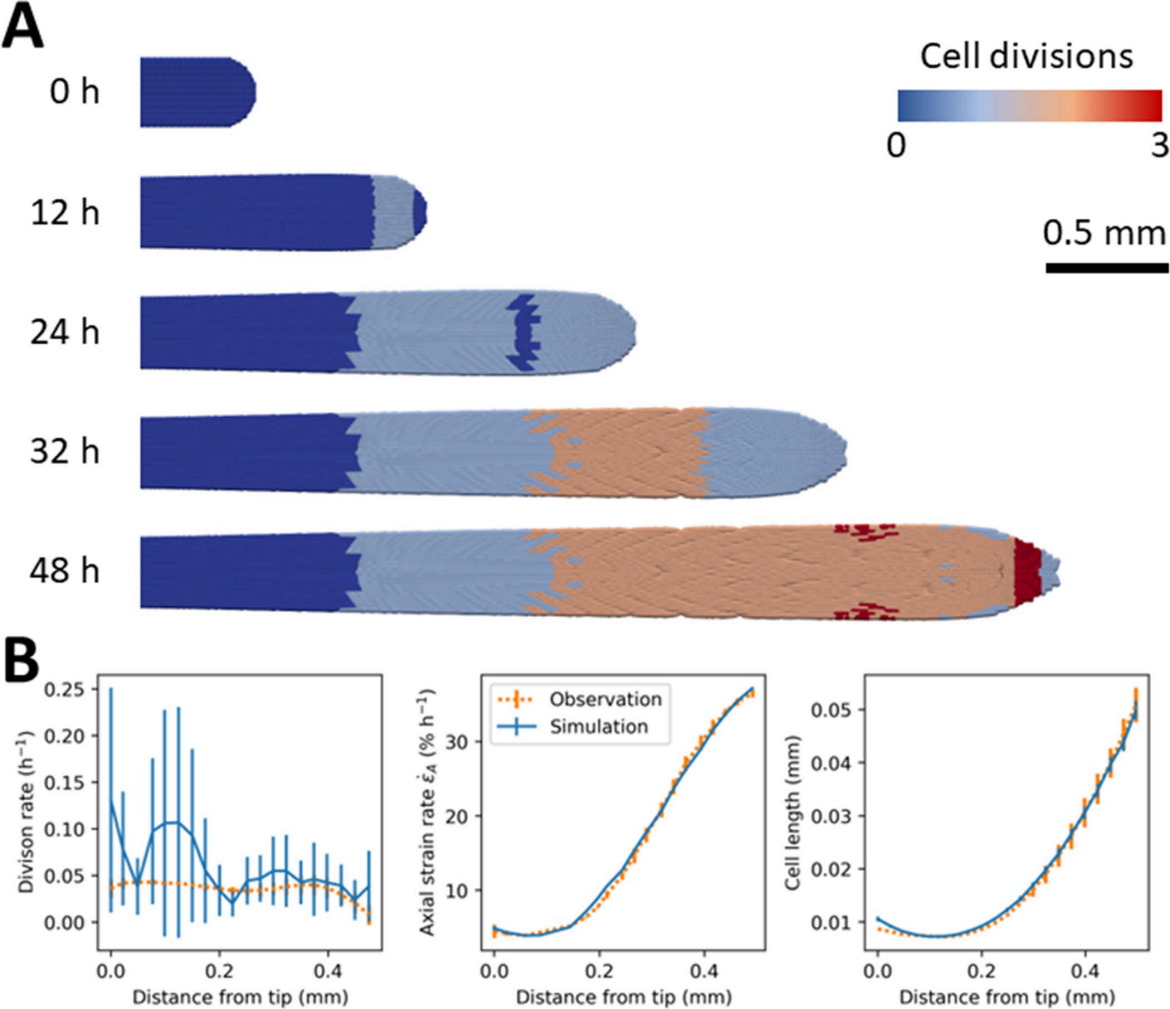
Simulation of unimpeded growth of Arabidopsis thaliana roots using axial strain rate data, cell length data and cell division rate data derived from [20]. (A) Simulations of the growth of a root for 50 hours. Colors indicate the number of cell divisions from the beginning of the simulation. Blue cells have not divided during the simulation. Red cells have divided three times. Simulations were initiated with particles distributed on a 2D cartesian lattice. (B) Comparison between model and experimental data. Comparison between experimental and SPH predictions for cell division rate (Left). Comparison between experimental and SPH predictions for axial strain rate (Center). Comparison between experimental and SPH predictions for cell lengths (Right). Symbols are mean ± SE over 40 consecutive time steps. Experimental observations are represented by orange dashed lines and numerical simulations by blue solid lines.

The cell division rate was better predicted in the root elongation zone than at the tip of the root (Fig 4B, left). Along the first 0.2 mm of root tissue, the simulated division rate was higher than in experimental observations. This could be due to the stochasticity of the data or an inadequate mathematical relationship between the cell division threshold $\bar{l}$ and the distance from the root tip. Our model also could have generated undesired cell divisions in the radial direction. All such deviations could be prevented in future version of the model using more detailed experimental data on cell divisions in the radial, tangential and axial directions [21]. In the root elongation zone, at a distance between 0.2 mm to 0.5 mm from the tip, the model showed better agreement with observation. The predicted division matched experimental data with R^2^~18%, RMSE~1.3×10^−3^. The variations of axial strain rate and cell length were predicted with higher accuracy (Fig 4, center and right) with R^2^ coefficient above 99% for both and RMSE being equal to 6.2×10^−7^ and 0.35, respectively.

The fitting of model to experimental strain rate data required the softening of tissues to increase from the root tip following a bell-shaped function described in Eq 6, second, with a maximum located at *x*_*g*_ = 0.32 mm, a slope *s*_*g*_ = 0.25, and constant *c*_*g*_ = 0.4. We found that the cell division threshold $\bar{l}$ followed a quadratic function described in Eq 6, third, with slope *s*_*d*_ = 45, a minimum located at *x*_*d*_ = 0.12 mm, and scaling coefficient *c*_*d*_ = 1. To maintain stable root diameter and tip shape, we found that the radial modulus *E*_*R*_ must increase smoothly following the sigmoid described in Eq 6, first, with an inflection point located at *x*_*e*_ = 0.32 mm, with slope *s*_*e*_ = 6, and constant *c*_*e*_ = 0.2 (Figure B in S1 text). The size of cells varied greatly along the first 0.6 mm of the root tip. The model used a smoothing length coefficient $\bar{H}=8$, which provided sufficient stability and computational efficiency to cover a ten-fold extension of cells. Larger cells were more difficult to include in a computationally efficient way because larger $\bar{H}$ values required for numerical stability would over-smooth dynamics at smaller scales.

Using these parameters, stability was obtained for simulations of long durations (S1 Video). For example, it was possible to predict the growth of roots for 50 hours. During this time, the root volume increased by a factor of 10 (Eq 4). 2D computations were performed in 12 minutes using a computer with 32 cores.

### 2.4 Root response to soil mechanical resistance

The final series of simulations were performed to explain how the mechanical properties of the roots of *Pisum sativum* (pea) change in response to increase of soil strength, using strain rate data from [15] and root radius data from [16]. The fitting of the model was achieved following the method detailed in section 4.5 of the material and methods where the softening coefficient (*λ*) is adjusted first to fit the strain rate data, the anisotropy of the tissue *E*_*R*_ is then fitted to root diameter data. There was no cell size data provided in these publications, so the critical cell length was adjusted arbitrarily (Table 1).

**Table 1:** Description of model parameters. When cases involve more than one set of parameters, differing parameters are listed into brackets.

Name	Description	Case 1	Case 2	Case 3	Units
*p*	Pressure differential	1	1	[0.5, 1]	MPa
*E*_*A*_	Axial Young modulus	1020	1020	1020	MPa
*E*_*H*_	Maximal radial Young modulus	15000	15000	15000	MPa
*ν*_*A*_	Axial Poisson modulus	0.06	0.06	0.06	
*ν*_*R*_	Radial Poisson modulus	0.3	0.3	0.3	
*x*_*e*_	Position of state transition	0.6	0.51	[2.5, 3]	mm
*s*_*e*_	Slope of sigmoid function	[0,0,5]	6	[1.3, 4]	mm^-1^
*c*_*e*_	Scale coefficient	[−1, 1, 0]	0.2	[0.26, 0.34]	
*ρ*_0_	Equilibrium density	1000	1000	1000	μg mm^−3^
*λ*_0_	Reference softening coefficient	0	4×10^6^	1.8×10^6^	μg mm^3^h^−1^
*x*_*g*_	Inflection of bell-shape function	−	0.32	[−0.4, −0.1]	mm
*s*_*g*_	Slope of bell-shape function	−	0.25	[2.5, 4.5]	mm
*c*_*g*_	Scale coefficient	−	0.1	[−1, 0.9]	
ℓ_0_	Reference cell size	0.01	0.01	0.08	mm
*x*_*d*_	Position of minimal division threshold	−	0.12	0	mm
*s*_*d*_	Slope of quadratic function	−	45	0	mm^-2^
*c*_*d*_	Scale coefficient	−	1.0	1.5	
*T*	Final time	8	100	100	h
$\bar{H}$	Smoothing length coefficient	2	8	4.5	

The model predicted well the 20% increase in root diameter in the mature part of the root in response to the change in pressure differential (*R*^2^>98%, *RMSE*<0.004 for *p* = 0.5 and 0.825 and *R*^2^>97%, *RMSE*<0.007 for *p* = 0.75). Strain rates from [15] were well predicted by the model (*R*^2^~86%, *RMSE*~1.6 for *p* = 0.987 and *R*^2^>97%, *RMSE*<0.78 for *p* = 0625). Discrepancies between experimental observations and model predictions occurred in the mature region of the root. These were due to the model accounting for changes in mechanical properties only at the root apical meristem and no softening was allowed beyond that point of the root.

**Fig 5:**
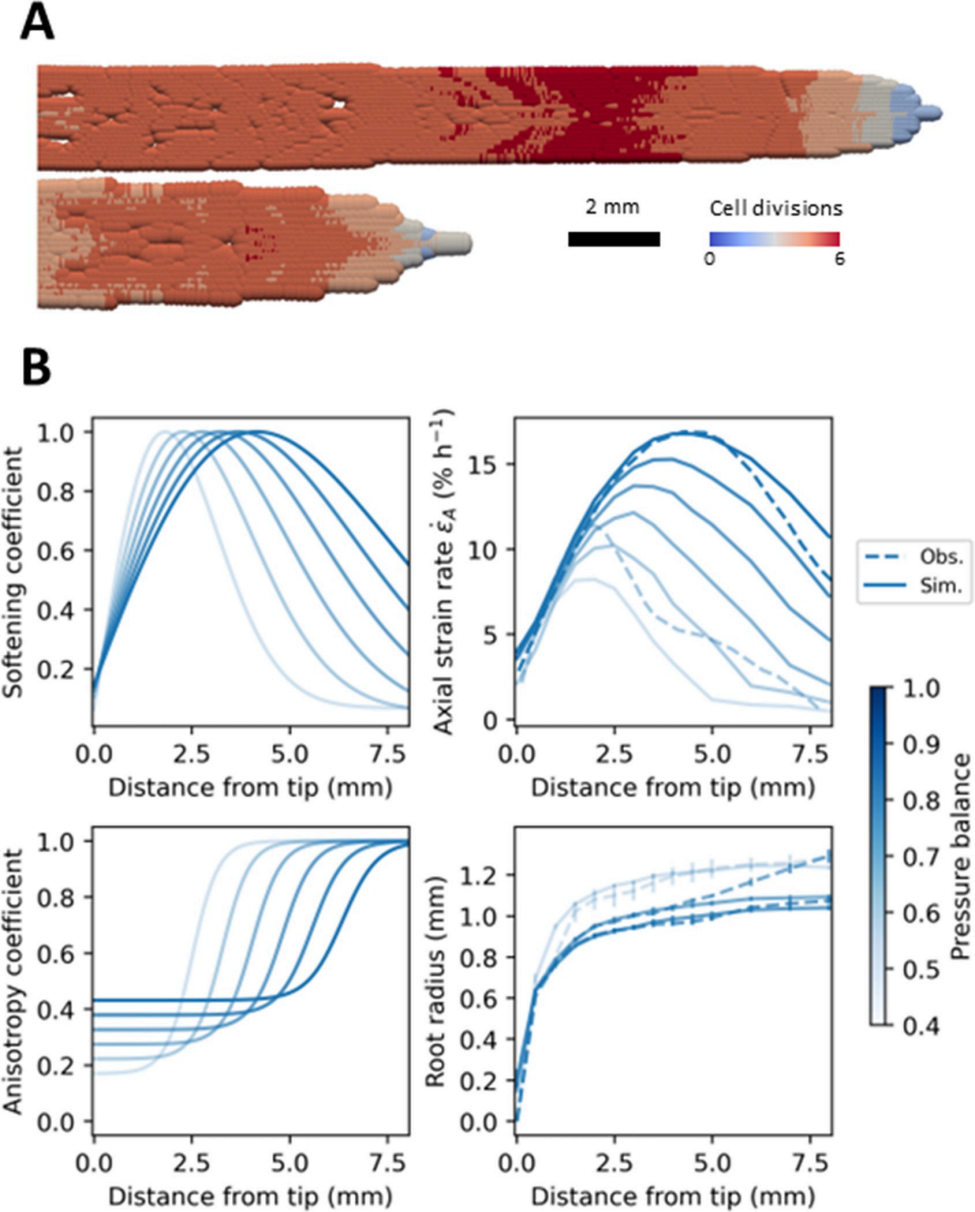
Simulation showing how the softening and anisotropy coefficients vary in the roots of Pisum sativum in response to increases in soil strength using strain rate data from [15] and root radius data from [16]. (A) Results of SPH simulation for a pressure differential of 1 (top) and 0.4 (bottom). Simulations were initiated with particles distributed on a 2D cartesian lattice. Only the mechanical parameters of the model were adjusted to data. The cell division rate was set to a constant threshold of 120 μm which produced reasonable average cell sizes and numerical stability. (B) To match experimental data, the softening coefficient λ was found to reach a maximum closer to the tip and tail off quicker for harder soils (top left). Such variations in the softening coefficient along the root allowed the SPH model to match the axial strain rate observed experimentally (top right). The radial modulus *E*_*R*_ was found to take smaller values at the tip but increased quicker to reach its maximum for harder soils (bottom left). These variations of radial modulus along the root allowed the SPH model to match the variations of root radius observed experimentally (bottom right). Experimental observations are represented by dashed lines and numerical solutions by solid lines. Increase in the pressure differential is represented from light blue to dark blue.

Along the first millimeter of root, the strain rate remained identical across the range of studied pressure differentials. The size of the root elongation zone however varied with soil strength. In loose soils, the size of the root elongation zone was larger, and the root axial strain rate was higher. The maximum axial strain rate was observed at larger distances from the root tip. In hard soils, the softening coefficient was found to increase closer to the tip and have a tail that declines faster with the distance from the tip (Fig 5A, top left). Growth parameters in Eq 6 depend on the pressure differential *p* using Eq 7 with parameters *x*_*g*_ = −0.4 mm, *s*_*g*_ = 4.5 and *c*_*g*_ = −1 for *p*_*g*_ = 0.987 MPa, and *x*_*g*_ = −0.1 mm, *s*_*g*_ = 2.5 and *c*_*g*_ = 0.9 for *p*_*g*_ = 0.625 MPa. Results of simulations showed the increased softening at the root tip was sufficient to predict accurately the growth of the tissue and the corresponding experimentally observed strain rates (Fig 5B, top right) where *p* = 0.4 MPa correspond to a hard soil and *p* = 1.0 MPa to a soft soil.

To predict the increase in root radius, the radial modulus *E*_*R*_ must also vary with the pressure differential *p*. The model fitted experimental data with parameters in Eq 6 with *x*_*e*_ = 3 mm, *s*_*e*_ = 1.3 and *c*_*e*_ = 0.34 for *p* = 0.825 MPa, and *x*_*e*_ = 2.5 mm, *s*_*e*_ = 4.0 and *c*_*e*_ = 0.26 for *p* = 0.5 MPa. These parameters resulted in a shift of the anisotropic transition closer to the root tip and a smoother transition through the root elongation zone. Close to the tip, the root tissue was also more isotropic in hard soil (Fig 5B, bottom left). Using these parameters, the SPH model could predict accurately the increase in root diameter observed experimentally (Fig 5B, bottom right, S2 Video).

## 3 Discussion

### 3.1 New framework for modeling root meristems at cell scale

There are only a handful of modeling approaches able to resolve biophysical interactions within a growing root at the cell scale. Early models for plant root tissue considered 1D continuous description [12,22] and have exposed the balance of forces acting on growing roots. Since then, models have included more detailed endogenous [23] and exogenous processes [24], but the use of one-dimensional descriptions remains limiting. For example, it is not easy to describe bending and buckling caused by rigid obstacles, or growth through paths of least resistance in soil [1]. The extension to higher dimensions proposed by [25] was met with limited success because the use of growth tensors requires many analytical calculations. Numerical techniques such as the Finite Element Method (FEM) have overcome these limitations and applications now include predictions of mechanical stress and failure zone in soil, water transport in roots, or studies of root gravitropic responses [16,26,24].

Cellular models have emerged subsequently as a mean to account for morphogenetic processes. The use of vertex-based approaches has proven particularly powerful to simulate complex developmental processes, including phyllotaxis, lateral root formation, cell differentiation patterns on leaves, and cell-cell communication [9]. Recent computational techniques couple vertex dynamics to cell volume and implement quasi-static approximations to improve the stability of computations. However, because each cell in the system must be described through a large number of vertices, computations are slow and difficult to parametrize and applications have largely focused on problems with reduced physical dimensions, e.g. outermost layers of cells, or using two-dimensional descriptions [26,27,28].

Particle-based approaches are relatively recent alternatives. Numerical approximations are less well characterized than finite element methods, and the calibration of numerical algorithms remains complex [29]. The approximations computed by the Smoothed Particle Hydrodynamics (SPH) method depend strongly on the particle distribution and the averaging domain defined by the smoothing length. When axial and radial strains are large, the distribution of particles becomes too heterogeneous, numerical errors increase and SPH simulations can result in undesired clogging, detachment of particles or cracks. Such effects could occasionally be observed in our simulations, e.g. Fig 5A, but their impact could be controlled to not affect model predictions.

This work presented a first-generation model which cannot predict all biological process that are observed experimentally. Associating the SPH particle with the cell brings constraints because the size of particles cannot be used to increase accuracy of numerical solutions, which in turn depends only on the kernel function. Also, large variations in cell sizes occur between the different parts of the root tip, which require a fine tuning of the smoothing length. The smoothing length required for stability is often determined by the region of the domain with the lowest density of particles, making simulations across the entire domain less accurate. Here we used simplified cell division rules that limited cell length in the growing roots. In the case of *Arabidopsis* roots, we fit the model of cell division rate to experimentally observed data on the first 500 μm from the tip but kept the cell division threshold $\bar{l}$ constant beyond 500 μm. This reduced the appearance of cracks but also overestimated the cell division rate in elongating cells further away from the root tip. The cell division rule was also simplistic in other aspects. The cell division was always symmetric, it did not account for cell types, and it was solely determined by cell position and size. The model was therefore unable to predict the root anatomy. A universal cell division rule has yet to be discovered [30,31,32,33], which limits current prospects of predicting accurately the root cellular architecture.

Since the first publication of the SPH method in 1977 [34,35] the field of research has progressed at a considerable pace with, for example, the introduction of anisotropic kernels to compute large strain, implicit and incompressible schemes to stabilize long-term simulations, corrected and symmetric formulations to manage numerical errors, and improved neighbor search for better efficiency [19]. Finally, communities are pushing for development of unified SPH framework using open Application programming interfaces [36]. This makes SPH methods very promising for application to plant morphogenesis. The SPH method proved amenable to high dimensional and multi-physics problems because kernel functions can easily be formulated in n-dimensions. Tests performed on two-dimensional domains can therefore be ported to three dimensional problems without the need for a new interpolation scheme. Also, higher spatial dimensions and unstructured grids increase the number of neighbors and reduces the occurrence of cracks and cell detachment, the latter being also an interesting feature when modelling cell detachment in the root cap, for example.

**Fig 6:**
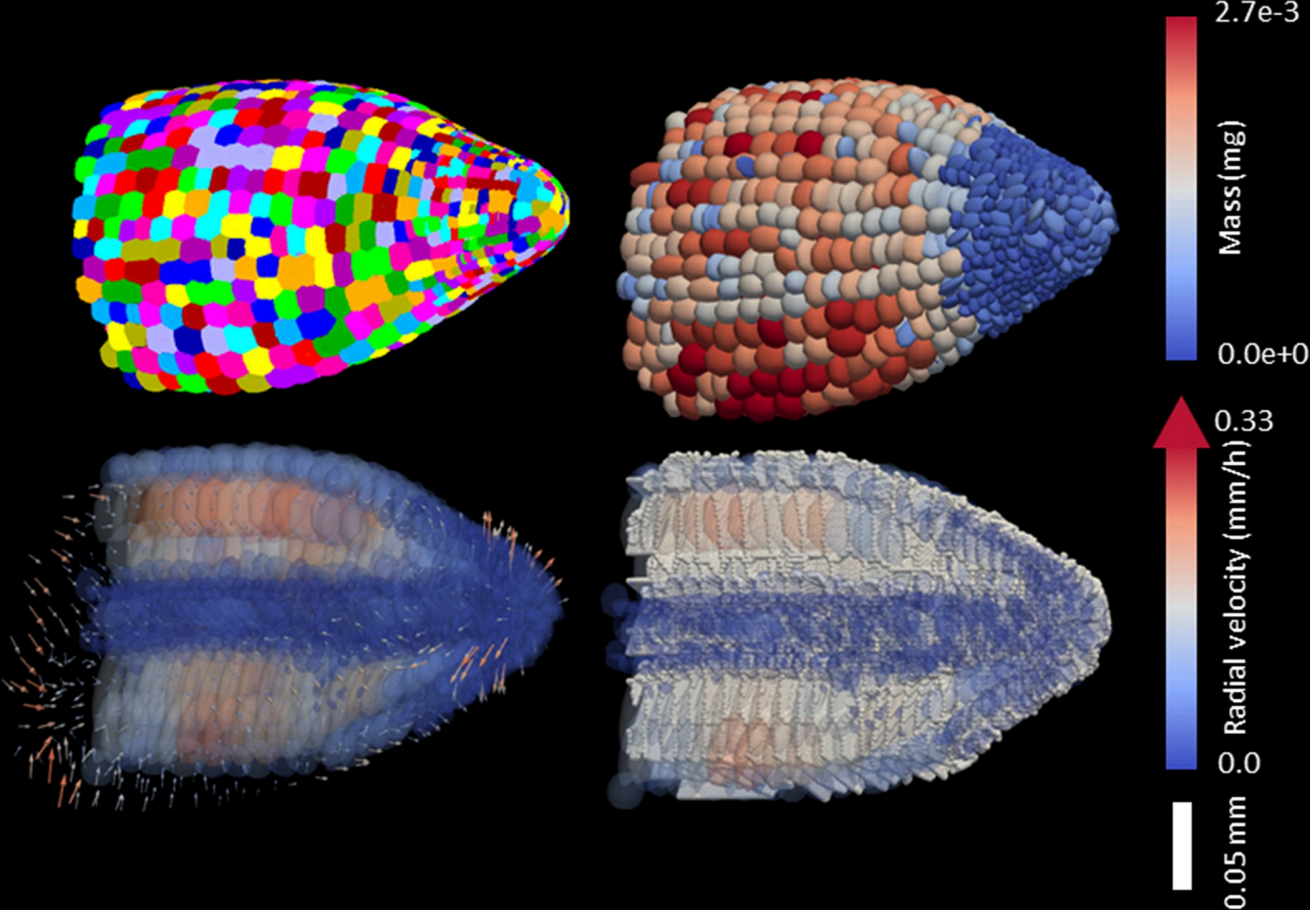
Future applications for SPH models in root developmental biology. Image processing pipelines currently available to analyze 3D live microscopy data can extract the geometrical properties of cells (top left). These were used to compute the shape matrix of particles *Q*_*i*_ and were used as input for SPH simulation (top right). The model then could compute the growth of plant roots, including cell size (bottom left, colored ellipsoids) and strain rate (bottom left, arrows show radial velocity). Kernel functions could then be used to compute cell walls (bottom right). Here, the root is represented either as a whole (top) or following a cross section (bottom).

Here, using SPH we have demonstrated the feasibility of computing root-soil interactions at cell scale across the entire organ. The technique allowed us to associate numerical particles with biological cells, preserving the shape, size, and cellular architecture of the root with a minimal number of degrees of freedom (Fig 1B). Particles describe the interior of the cell, and cell walls can be reconstructed from the kernel function. Flexible numerical schemes allowed computation of thousands of cells using a modern workstation [37], and the method could compute whole root meristems in three dimensions (Fig 6, S3 and S4 Videos). Simulations could be performed using images of the root anatomy with limited image processing because only basic shape descriptions for the cells are needed as an input [7].

### 3.2 Root strategies to overcome mechanical resistance from the soil

Using our SPH model, we could predict the growth of a root from its biophysical parameters (Fig 4). The model did not include turgor pressure in the response because data shows only limited increases in turgor pressure are observed in response to increased impedance from the soil [13,14,15]. The model focused on the softening of the tissue and assumed a constant turgor pressure. However, recent studies using Atomic Force Microscopy have revealed that turgor pressure in plant tissues is heterogeneous [38]. Such variations have been attributed to cell size and growth rate but may also play a role to overcome external forces from the soil. Future model development could include a complete poromechanical description of the tissue to include the regulation of turgor pressure in the tissue. To explain growth rates observed in soils which penetrometer resistance exceed turgor pressure, we also chose the simplifying assumption proposed in [39] that the pressure perceived by the plant is a fraction of the penetrometer resistance of the soil.

The model revealed how tissue mechanical properties may be rearranged when soil strength is increased. We first observed that pressure from the soil alone was not able to predict the morphology of roots growing in hard soil. Plant roots are known to reduce the size of their meristem but also to maintain the axial strain rate at the root apex. In soft soil, the root elongation zone is large and the root radius rather thin whilst in hard soil, it is the reverse situation, with roots showing increased diameter and axial strain rate concentrated at the tip. The observation was made in response to mechanical impedance from the soil but is also observed during water stress [40]. To predict such responses, tissue softening must reach a maximum closer to the root tip to overcome soil resistance, likely with an increase in softening at the tip of the root but increased turgor pressure may cause the actual softening of tissue to be lower than predicted. This occurs in a small region of the root tip, because excessive softening could lead to mechanical weakness, as was observed for example when roots were exposed to axial forces [1]. The softening of tissue in the root tip is also associated with the shortening of the zone where softening occurs, and an increase in anisotropy of the tissue. Such response may reduce the susceptibility of the root to buckling [10]. Realistic growth responses were obtained without the need for the maximum of the softening coefficient to increase. This could indicate that excessive softening of the tissue may be detrimental. For example, it could affect the mechanical stability of the root tissue [41].

Root diameter was also observed to increase by 20% to 60% when penetrometer resistance is doubled [16]. Axial forces alone can explain radial growth of the root due to incompressibility. For example, an axial mechanical stress of 1 MPa with a Young’s modulus of 100 MPa and a Poisson coefficient of 0.3 explains a radial strain of 0.3%. To match observations, the model also requires a change in the radial modulus. The tissue must become more mechanically isotropic at the root tip and more anisotropic in the root elongation zone. This response is also consistent with requirements for mechanical stability when external pressures increase. Increased isotropy and cell wall softening at the root tip generates soil cavity expansion, a process which has been shown to reduce the axial load on the root tip [3], and results in the larger diameter measured experimentally. In the root elongation zone however, the strengthening of the tissue in the radial direction may reinforce the root against axial mechanical stress, and together with the increase in diameter may prevent the root from buckling.

### 3.3 Models for the next generation of root microscopy data

Data available to quantitatively characterize root processes is growing rapidly. Live microscopy in soil-like conditions has greatly improved with the development of artificial soils [42,43]. Microfabrication techniques now facilitate the construction of microcosms with a high degree of control on growth conditions [44], and this proved a particularly promising approach to acquire root kinematics and cell division data suitable for the calibration of models [21]. The emergence of light sheet microscopy is democratizing the use of instruments built in-house for larger living samples [45], and techniques such as Brillouin scattering are beginning to reveal how cell wall mechanical properties and cell wall softening vary across plant root tissues [46]. Available techniques to quantify growth, morphology and anatomy from roots grown in natural soils has also improved drastically with techniques such as X-ray tomography, MRI and neutron tomography, and Laser Ablation Tomography [47]. Microfluidics and robotics [48] now enable fast three-dimensional reconstruction of the root architectures at anatomical and whole plant level. Likewise, algorithms for processing of image data are improving. Software enables larger samples to be reconstructed from a myriad of views [49] and the automated extraction and classification of features [50]. The availability of software resources to analyze and process root microscopy data is also greatly expanding thanks to repositories for open-source software [51] and microscopy data [52]. The emergence of standards for data formats stimulates portability and communication between software with reduced efforts needed in software development [53,54].

Biophysical models are becoming a critical component of these software pipelines. Models could help forecast root performance in field conditions from quantitative traits now delivered by modern microscopes and live imaging facilities. Currently, mathematical models able to exploit information contained in large imaging data sets are limited, and this paper has demonstrated the potential for particle-based approaches to bridge this gap. The technique requires minimal description of the anatomic structure of the roots. It is computationally efficient (we could compute three-dimensional simulation of a root during a period of 55 hours, increasing the number of cells from 45,000 to 200,000 for an increase in length of 25 mm), and can easily be integrated into pipelines for the simulation of realistic and complex scenarios (Fig 6). Results presented here showed that the model and simulation pipeline can reveal the complex responses of roots to the strengthening of soils. The next step now is to confirm the results with a broad range of root anatomy and adequate field validation. This would further consolidate the potential of image-based modeling to assist experimental research.

## 4 Material and methods

### 4.1 Tissue level dynamics

Tissue dynamics are modeled using the framework of continuum mechanics for finite strain. This framework describes the conservation of mass and momentum such that, in the absence of external forces,

$$\begin{array}{ll} \frac{D\rho }{Dt} & =-\rho \nabla \cdot u+\gamma (\rho ),\\ \frac{Du}{Dt} & =-\frac{\nabla (P(\rho )+\tau )}{\rho },\\ \frac{D\tau }{Dt} & ={\Lambda S}^{-1}\dot{\varepsilon }, \end{array}$$ (1)

where $\frac{D}{Dt}$ denotes the material derivative. The model equations link the particle velocity *u* (mm h^−1^) to the tissue density *ρ* (μg mm^−3^) and to the Cauchy stress tensor *σ* (MPa), also referred to as mechanical stress, given by the sum of the isostatic pressure *P* and a deviatoric component *τ* (shear stress). The strain rate $\dot{\varepsilon }=\frac{1}{2}(\nabla u+\nabla u^{T})$ characterizes the growth of the tissue. The matrix **Λ** selects the deviatoric contribution of the mechanical stress and is given by $\Lambda =I-\frac{1}{3}{\left(1,1,1,0,0,0\right)}^{T}(1,1,1,0,0,0)$.

Growth is modeled through the source term *γ* in the Eq 1 given by

$$\gamma (\rho )=\lambda {\left(\frac{\rho_{0}}{\rho }-1\right)}^{+}.$$ (2)

Here *γ* (μg mm^−3^ h^−1^) is expressed as a function of the difference between the equilibrium density *ρ*_0_ and the actual density *ρ*. Hence, the model incorporates a softening mechanism assuming that the deposition of the tissue material is instantaneous. The coefficient *λ* (μg mm^−3^ h^−1^) controls the tissue softening. Only the positive part is used in Eq 2 to represent the irreversibility of the extension of the cells. A constitutive equation relating the volumetric strain to isostatic pressure is given in a weakly compressible setting [19],

$$P(\rho )=K\left(\frac{\rho }{\rho_{0}}-1\right),$$ (3)

with *K* (MPa) being the bulk modulus. Eqs 2 and 3 describe an irreversible growth process with a densification-relaxation process, similar to the Lockhart and Ortega models [7,22].

To account for the anisotropic growth of root tissues, the material is assumed to be transversely isotropic in the axial direction, and the compliance tensor **S** is defined using the Voigt notation,

$$S=\left(\begin{array}{cccccc} \frac{1}{E_{L}} & -\frac{\nu_{L}}{E_{L}} & -\frac{\nu_{L}}{E_{L}} & 0 & 0 & 0\\ -\frac{\nu_{L}}{E_{L}} & \frac{1}{E_{R}} & -\frac{\nu_{R}}{E_{R}} & 0 & 0 & 0\\ -\frac{\nu_{L}}{E_{L}} & -\frac{\nu_{R}}{E_{R}} & \frac{1}{E_{R}} & 0 & 0 & 0\\ 0 & 0 & 0 & \frac{2(1+\nu_{R})}{E_{R}} & 0 & 0\\ 0 & 0 & 0 & 0 & G & 0\\ 0 & 0 & 0 & 0 & 0 & G \end{array}\right),$$ (4)

where *E*_*L*_ and *E*_*R*_ are axial and radial moduli, respectively, *ν*_*L*_ and *ν*_*R*_ are the Poisson moduli in the tangential and radial planes, respectively, and *G* is the shear modulus in the axial direction. Finally, using Eq 4, we obtain the expression for the bulk modulus

$$K=\frac{1}{S_{11}+S_{12}+S_{13}+S_{21}+S_{22}+S_{23}+S_{31}+S_{32}+S_{33}}.$$

### 4.2 Cell level dynamics

The individual cells in the tissue are described by particles carrying state variables such as mass, physical dimensions, and velocity. The cells are described as ellipsoids (Fig 1B, bottom), by a symmetric positive definite matrix *Q* = (*Q*_*ij*_)_*i*,*j* = 1,2,3_

The length of the cell’s main axes is obtained through the computation of *Q*’s eigenvalues and eigenvectors,

$$Q=V\left(\begin{array}{ccc} \zeta_{1} & 0 & 0\\ 0 & \zeta_{2} & 0\\ 0 & 0 & \zeta_{3} \end{array}\right)V^{T},$$

where *ζ*_*i*_ are the eigenvalues and *V* is the matrix composed of the corresponding eigenvectors (Fig 2B). By convention, *ζ*_*i*_ are ordered such that *ζ*_1_>0 is the smallest eigenvalue.

To determine the growth and division of a cell, the shape of the ellipsoid is evaluated at discrete time steps noted *t*^*n*^. The growth of the ellipsoid is updated at every time step *t*^*n*+1^, based on the velocity gradient ∇*u* so that the deformed ellipsoid is defined by

$$Q^{n+1}=(I-\Delta t\nabla u^{T})Q^{n}(I-\Delta t\nabla u),$$

where Δ*t* = *t*^*n*+1^ −*t*^*n*^. A cell division occurs when one axis of the ellipsoid reaches the cell division threshold $\bar{l}$. The cell length ℓ is expressed as a function of the length of the principal cell axis $l=2\sqrt{\frac{1}{\zeta_{1}}}$ and division occurs when $l>\bar{l}$. Cell division generates two equal particles on each side of the division plane along the longest axis of the mother particle, at half radius distance (Fig 2C). Following division at *t* = *t*^*n*^, the shape of the divided cell is given by

$$Q^{n}=V\left(\begin{array}{ccc} 4\zeta_{1}^{n} & 0 & 0\\ 0 & \zeta_{2}^{n} & 0\\ 0 & 0 & \zeta_{3}^{n} \end{array}\right)V^{T}.$$

Since the division plane occurs perpendicular to the longest axis, it is consistent with energy minimization principles used in cell division models [30]. This same division rule was applied across all cell types within the tissue. The cell division threshold $\bar{l}$ was kept sufficiently low to avoid large cells producing cracks. This is a simplification of the growth processes observed experimentally in plant roots with deviations from experimental data occurring also due to cell division rules changing as a function of the plane where the division occurs, the type of cell, or due to root interactions with the surrounding environment.

### 4.3 Mechanical impedance by soil

To simplify computations, mechanical resistance from the soil is modelled through application of a uniform pressure on the root tissue. The pressure applied on the root is introduced in the equation for momentum conservation (Eq 1, second) and is modified as

$$\frac{Du}{Dt}=-\frac{\nabla (P(\rho )+p+\tau )}{\rho }.$$ (5)

Here *p* is the difference between turgor pressure and soil pressure perceived by the root. Roots have been shown to perceive only a fraction of the mechanical resistance of the soil that is measured by penetrometer test. Explaining factors are reduced friction and growth through path of least resistance [1]. Here, the mechanical resistance of the soil was assumed to be a fourth of the pressure recorded by penetrometer test [39].

### 4.4 Smoothed Particle Hydrodynamics implementation

Unlike classical Finite Element or Finite Volume Methods, mesh-free methods use particles without a predefined topology of neighbors. The SPH approximation of a variable, denoted by 〈∙〉, is calculated from the weighted interactions between neighboring particles. The weighting is obtained with the kernel function (Fig 2). The kernel function is related to the cell size through the smoothing length coefficient $\bar{H}$, see Method A in S1 Text. The time evolution of state variables is computed with a prediction-correction algorithm [55]. It computes first a prediction of the evolution at the half time step, and then corrects the estimation at the complete time step. The state variables of particle *i* at half time step $t=t^{n+\frac{1}{2}}$ are computed as

$$\begin{array}{ll} \rho_{i}^{n+1/2} & =\rho_{i}^{n}+\frac{\Delta t}{2}\left\langle \frac{D\rho_{i}^{n}}{Dt}\right\rangle ,\\ u_{i}^{n+1/2} & =u_{i}^{n}+\frac{\Delta t}{2}\left\langle \frac{Du_{i}^{n}}{Dt}\right\rangle ,\\ x_{i}^{n+1/2} & =x_{i}^{n}+\frac{\Delta t}{2}u_{i}^{n},\\ \tau_{i}^{n+1/2} & =\tau_{i}^{n}+\frac{\Delta t}{2}\left\langle \frac{D\tau_{i}^{n}}{Dt}\right\rangle . \end{array}$$

Then at the complete time step *t* = *t*^*n*+1^, state variables are computed as

$$\begin{array}{ll} \rho_{i}^{n+1} & =\rho_{i}^{n}\frac{2-\delta^{n+1/2}}{2+\delta^{n+1/2}},\;\mathrm{with}\;\delta^{n+1/2}=-\frac{\Delta t}{\rho_{i}^{n+1/2}}\left\langle \frac{D\rho_{i}^{n+1/2}}{Dt}\right\rangle ,\\ u_{i}^{n+1} & =u_{i}^{n}+\Delta t\left\langle \frac{Du_{i}^{n+1/2}}{Dt}\right\rangle ,\\ x_{i}^{n+1} & =x_{i}^{n}+\Delta t\frac{u_{i}^{n+1}+u_{i}^{n}}{2},\\ \tau_{i}^{n+1} & =\tau_{i}^{n}+\Delta t\left\langle \frac{D\tau_{i}^{n+1/2}}{Dt}\right\rangle ,\\ Q_{i}^{n+1} & =\left(I-\Delta t\langle {(\nabla u_{i}^{n+1/2})}^{T}\rangle \right)Q_{i}^{n}\left(I-\Delta t\langle \nabla u_{i}^{n+1/2}\rangle \right). \end{array}$$

Details on SPH approximation for $\left\langle \frac{D\rho_{i}^{n}}{Dt}\right\rangle ,\left\langle \frac{Du_{i}^{n}}{Dt}\right\rangle ,\left\langle \frac{D\tau_{i}^{n}}{Dt}\right\rangle$, and $\left\langle \nabla u_{i}^{n}\right\rangle$ are specified in Method A in S1 text. In short, the operator 〈⋅〉 is a weighted sum over a set of neighboring particles, with the weight given by the kernel function evaluated on the particle of interest. The kernel function must therefore bring more weight to close particles than to the particles further away. For this reason, the kernel is a strictly decreasing function with the distance from its particle, and the smoothing length $\bar{H}$ is used to determine the distance over which it can affect its neighbors. Several functions exists and lately, the Wendland kernel is preferred for its intrinsic stability properties [19].

In contrary to a vertex-based model, field variables are defined only once for each cell. This reduces the number of unknown in the system and make the approach computationally efficient. It is impossible however to compute subcellular processes explicitly, especially those taking place in the cell wall. Modelling the growth of a root hair cell with a SPH approach would therefore not be feasible.

Computations were performed using initial particle distributions either extracted from microscopy data or generated on a Cartesian lattice. The initial geometries consisted of a rectangle (2D) or cylinder (3D), merged with a half disk, or half sphere, respectively. In the absence of specific hypotheses on cell size distribution, particles were initiated as spheres with initial diameter ℓ = ℓ_0_. At the base of the domain, particles were fixed and prevented from moving to represent the connection with the plant body. Particle velocity and mechanical stress were assumed to be zero as initial conditions. Duration of computational time was determined so that the model has reached a steady state. The Courant-Friedrichs-Lewy condition controlled the stability and the time step *Δt* was set to 0.9.

The code was implemented in C++ (compiled with v140 tool set on Windows and gcc 8.4 in Linux) and source files are available at https://github.com/MatthiasMimault/RootSPHysicsV2_DB.

### 4.5 Parameterization of the model

To adjust the model parameters to fit experimental data, we considered three types of parameters. The parameters that affect the anisotropy of the tissue along the root are denoted with a subscript *e* and affect the radial modulus *E*_*R*_. The parameters that affect growth are denoted with the subscript *g* and affect the softening coefficient *λ*. The parameters that affect cell division are denoted with the subscript *d* and affect the cell division threshold $\bar{l}$. *E*_*R*_, *λ* and $\bar{l}$ were also assumed to depend on the soil resistance and the axial position *x* along the root:

$$\begin{array}{c} E_{R}(x)=E_{A}+(E_{H}-E_{A})(c_{e}+\frac{1-c_{e}}{1+e^{-s_{e}(x-x_{e})}}),\\ \lambda (x)=\lambda_{0}\left[\frac{1+c_{g}}{2}+\frac{\left(1-c_{g}\right)}{2}\frac{\left(x-x_{g}\right)}{s_{g}}e^{0.5-\frac{{\left(x-x_{g}\right)}^{2}}{2s_{g}^{2}}}\right],\\ \bar{l}(x)=l_{0}(c_{d}+s_{d}{\left(x-x_{d}\right)}^{2}). \end{array}$$ (6)

The first relationship in Eq 6 defines the variations of the radial modulus *E*_*R*_ along the root and causes growth to be isotropic at the tip and anisotropic at the base of the root with *E*_*H*_ being the maximal radial modulus. The second relationship describes the variations of the softening coefficient *λ* along the root and affects the size and kinematics of the growth zone. Finally, the third relationship describes the variations of the cell division threshold $\bar{l}$ along the axial direction of the root and defines the kinematics of the cell division zone. Model parameters used in numerical simulations are specified in Table 1. The development of the root is therefore fully determined by the set of parameters $\xi =(c_{e},x_{e},{s_{e},c}_{g},s_{g},x_{g},c_{d},s_{d},x_{d})$.

Model parameters *ξ* were estimated to fit experimental data for the strain rate, the cell length, and the root diameter. Using optimization algorithm was challenging computationally, therefore, we made several assumptions to estimate model parameters. First, the softening coefficient and Young’s modulus are both linked to growth of the tissue through Eqs 2 and 3, but because only data on growth was provided in experimental studies the focus is first on fitting the softening coefficient. Therefore, we first fixed the axial Young’s modulus to a reasonable value appropriate for a biological tissue, and we focused on identifying growth parameters from data.

To obtain a crude estimate for the parameters *E*_*H*_, *λ*_0_, and *l*_0_, which control the magnitude of the variations in strain rate, radius, and cell length in the root meristem, we made trial and error simulations with root tissues having constant properties along the growth zone. For the critical length at division, we observed for example that the critical length is approximately 1.5 the mean cell length. In the next step, to make the model fit more closely the experimental data, we used the geometrical features of the functions in Eq 6, which link directly to features in experimental curves and are independent from one another (Figure B in S1 text). In all three functions in Eq 6, the parameter *x* represents the location of the transition from the tip to the mature part of the root (inflection point in the root diameter *x*_*e*_, maximum axial strain rate *x*_*g*_, and minimum of the cell length *x*_*d*_), the parameter *s* represents the slope or stiffness of the transition and the parameter *c* controlled the minimum value of the curve. Once the functions were adequately centered, a fine adjustment of *E*_*H*_, *λ*_0_ and *l*_0_ was made. In this case, we observed the relationship between the parameters and the magnitude of the model output and changed the parameter proportionally to the deviations observed between experiments and model predictions. On occasion, the last steps were repeated to correct for small deviations between model outputs and experimental data.

To determine how root growth adapts to soil conditions, we examined whether the radial modulus and the softening coefficient functions vary as a function of the pressure differential *p* (Eq 5). To test this hypothesis, the parameters *ξ* were assumed to vary with the pressure differential using the following linear interpolation,

$$\xi (p)=\theta (p)\xi_{1}+(1-\theta (p))\xi_{2}.$$ (7)

Here, *θ* is a linear function such that *θ*(*p*_1_) = 1 and *θ*(*p*_2_) = 0. To parameterize the model, it is therefore sufficient to estimate the parameter vector *ξ*_1_ for growth in soft soil conditions *p* = *p*_1_, and the parameter vector *ξ*_2_ for growth in hard soil when *p* = *p*_2_.

### 4.6 Case 1—Tissue anisotropy and the morphology of the root tip

A remarkable property of developing roots is their ability to maintain their shape and size during growth. To investigate the ability of our model to exhibit such behavior, we performed simulations considering three possible expressions for the radial modulus *E*_*R*_ in Eq 6. Constant isotropic properties were modeled using *c*_*e*_ = −1 and *s*_*e*_ = 0. Constant anisotropic properties were modeled using *c*_*e*_ =1 and *s*_*e*_ = 0. Balanced properties were modeled using parameters shown in Table 1. To assess the ability of the model to correctly predict anisotropy, we considered the case of constant mechanical stress in an anisotropic material so that the axial and radial strain components can be determined analytically.


$$\varepsilon_{A}(x)=\frac{1-\nu_{A}}{E_{A}},\;\varepsilon_{R}(x)=\frac{1}{E_{R}(x)}-\frac{\nu_{A}}{E_{A}}.$$ (8)

The analytical solutions of the model can then be compared to numerical solutions with strain components estimated as the difference between the size of the tissue at equilibrium and the size of the tissue at the start of the simulation.

### 4.7 Case 2—Growth in unimpeded conditions

In the second study case, we examined the ability of the model to predict the unimpeded growth of the root. The model was adjusted to the data proposed by [20] that were extracted using WebPlotDigitizer (https://automeris.io/WebPlotDigitizer/). The study focuses on the growth of the roots of *Arabidopsis thaliana* 6 days after planting. The data available consist of cell length, axial strain rate and division rates, and how these variables vary as a function of the distance from the root tip. The data were obtained by live microscopy and image analysis followed by kinematics analysis [56].

For the model to accurately predict growth kinematics parameters, we adjusted the radial modulus, the softening parameter, and the cell division threshold in Eq 6. We considered the first 0.5 mm of the root tip because it is where most cell division occurs. All simulations here were performed in a 2D setting with particles initially distributed on a Cartesian lattice.

### 4.8 Case 3—Growth in impeded conditions

In the final study case, we examined how the root biophysical parameters vary as a function of soil resistance. The model was adjusted to the data from two different studies on *Pisum sativum* plants (Pea). First, the axial strain rate of growing tissues was obtained from plants growing in soils of different mechanical resistance [15]. The data available consist of the axial strain rate as a function of the distance from the tip for roots grown in soil at two different mechanical resistance. The data were obtained by live microscopy and image analysis, followed by kinematics analysis [56]. A different study was used to obtain data on the changes in root diameter as a function of soil resistance [16]. Data points were extracted using WebPlotDigitizer. In our studies, the pressure differential *p* was determined as the difference between a turgor pressure of 1 MPa and one fourth of the measured penetrometer resistance, so that *p* varied from 1 (soft soil) to 0.5 (hard soil). In this case, only the mechanical parameters of the model were adjusted to data. The cell division rate was set to a constant threshold of 120 μm and ensured uniform distribution of particle across the root tissue (Table 1). All simulations here were performed in a 2D setting with particles initiated on a 2D cartesian lattice.

### 4.9 Data processing and segmentation

To compute statistics from SPH simulations, particle data were aggregated into bins of equal lengths. For a given time step, particles located in the same bin were used to compute the mean and the standard error (SE). The division rate was computed as the ratio between new particles and old particles during the time step. Mean and standard error were computed for each bin at 40 different time steps. The fit between simulations and experimental data was evaluated with the Relative error (*RE*), the Pearson coefficient of determination (*R*^2^) and the Rounded mean square error (*RMSE*), computed with the *sklearn* package version 0.24.1 [57] in python (version 3.8.8).

To perform computations on a realistic cellular architecture we used volume datasets acquired with confocal laser scanning microscopy and processed with MorphoGraphX software [54]. Results of the image analysis (.csv files) were used as input for SPH computations. The files contained a unique cell identifier, spatial position, and ellipsoid approximation for each cell. Results of SPH computations are exported as.vtk and.csv files to be visualized using Paraview [58]. Cell walls were rendered using local minima search algorithm (find_peaks, SciPy, version 1.6.2 [59]) applied to the kernel function potential (Fig 2).

## Supporting information

S1 text **Method A**. Detailed description of SPH theory. **Figure A**. Anisotropy coefficient distribution for the study of tissue anisotropy and the morphology of the root tip (Case 1). **Figure B.** Division threshold, softening coefficient, and anisotropy coefficient for the simulation of the development of *Arabidopsis thaliana* roots in unimpeded conditions (Case 2).
(PDF)

S1 Video Unimpeded root growth with a Cartesian lattice of cells as initial condition. Colors here and in the following videos indicate the number of cell divisions.
(MP4)

S2 Video Effect of soil impedance on growth with two different pressures differentials applied to a root with a Cartesian lattice of cells as initial condition. (MP4)

S3 Video Three-dimensional simulation of root growth with a Cartesian lattice of cells as initial condition. Computation predicted root elongation for a duration of 55 hours. The computation initiated with 45 000 cells and finished with 200 000 cells, modeled an elongation of 25mm and eight generations of cell division.
(MP4)

S4 Video Root growth against an obstacle with a Cartesian lattice of cells as initial condition. Computation predicted root elongation and subsequent buckling against a tilted rectangular obstacle for a duration of 30 hours against. The computation initiated with 2000 cells and finished with 4 500 cells, modeled an elongation of 15mm and three generations of cell division.
(MP4)
