# Supplementary material for: Particle-based model shows complex rearrangement of tissue mechanical properties are needed for roots to grow in hard soil
Source: PLoS Comput Biol. 2023 Mar 7;19(3):e1010916. doi: 10.1371/journal.pcbi.1010916 (PMC10072375; doi:10.1371/journal.pcbi.1010916)

S1 text

Method A

The estimation of a state variable f across the root tissues, for example Cauchy stress or velocity, is approximated by the following integral at a location $\mathbf{x} = (x, y, z) \in \mathbb{R}^3$

$$\langle f(\mathbf{x}) \rangle_h = \int_\Omega f(\mathbf{x}') W(|\mathbf{x} - \mathbf{x}'|, h) \mathrm{d}\mathbf{x}', \quad (1)$$

where the kernel W is a positive, monotone decreasing function of the interaction distance between two particles $|\mathbf{x} - \mathbf{x}'|$, and the smoothing length h controls the size of the interaction region. The kernel W satisfies for $\mathbf{x} \in \mathbb{R}^3$

$$\lim_{|h| \to 0} W(|\mathbf{x} - \mathbf{x}'|, h) = \delta(\mathbf{x} - \mathbf{x}'),$$
$$\int_\Omega W(|\mathbf{x} - \mathbf{x}'|, h) \mathrm{d}\mathbf{x}' = 1,$$
$$\int_\Omega \nabla W(|\mathbf{x} - \mathbf{x}'|, h) \mathrm{d}\mathbf{x}' = 0.$$

The SPH method computes (1) by a summation over a set of discrete data points corresponding to particles. Thus, the approximation f_i of the function f at the particle i located at $\mathbf{x}_i$ results from the following sum over all the particles j in the neighborhood

$$\langle f_i \rangle_h \approx \sum_j f_j \frac{m_j}{\rho_j} W(r_{ij}, h),$$
$$\langle \nabla f_i \rangle_h \approx \sum_j f_j \frac{m_j}{\rho_j} \nabla W(r_{ij}, h),$$

where $r_{ij} = |\mathbf{x}_i - \mathbf{x}_j|$, m_j is the mass and ρ_j is the density of the particle j.

In roots, the distribution of cells and their size vary greatly with time, and the classical SPH precision suffers from heterogeneous distributions. To mitigate these effects, we implement the SPH with anisotropic kernels and gradient correction. To account for anisotropic volume changes, the smoothing length used to compute the contribution of a particle j on a particle i is denoted h_{ij} and defined as the radius of the smoothing ellipsoid $\mathbf{H}$ in the direction r_{ij}. $\mathbf{H}$ dimensions are proportional to the cell ellipsoid with a factor $\bar{H}$. We refer to the review of Liu and Liu [2], for the derivation of the anisotropic kernel. The corrected gradient formulation introduced by Zhang and Liu [3] follows

$$\tilde{\nabla} W(r_{ij}, h_{ij}) = \nabla W(r_{ij}, h_{ij}) \odot \left[\sum_k \frac{m_k}{\rho_k} (\mathbf{x}_k - \mathbf{x}_i) \odot \nabla W(r_{ik}, h_{ik}) \right]^{-1},$$

where i and j label two different particles, k labels the particles in the neighborhood of i, and $\odot$ denotes the component-wise product. Here, h_{ij} is the radius of the ellipsoid $\mathbf{H}$. The velocity gradient of particles is expressed as

$$\langle \nabla u_i \rangle = \sum_j \frac{m_j}{\rho_j} (u_j - u_i) \tilde{\nabla} W(r_{ij}, h_{ij}).$$

The variation of mass and momentum is approximated by

$$\left\langle \frac{\mathrm{D}\rho_i}{\mathrm{D}t} \right\rangle = \sum_j m_j (u_j - u_i) \cdot \tilde{\nabla} W(r_{ij}, h_{ij}) + \gamma_i, \tag{2}$$

$$\left\langle \frac{\mathrm{D}u_i}{\mathrm{D}t} \right\rangle = \sum_j m_j \left(\frac{\sigma_i + \sigma_j}{\rho_i \rho_j} + \Pi_{\alpha,ij} \mathbb{I} \right) \cdot \tilde{\nabla} W(r_{ij}, h_{ij}), \tag{3}$$

with $\sigma_i = (P(\rho_i) + p_i)\, \mathbb{I} + \tau_i \in \mathcal{M}_{3\times 3}(\mathbb{R})$. The artificial viscosity term $\Pi_{\alpha,ij}$ with $\alpha \in [0, 1]$ is described in [2] and is used to remove high frequencies oscillations. In our simulations,

we consider $\alpha = 0.6$. The deviatoric stress τ is computed using the Jaumann derivative [1], defined as

$$\left\langle \frac{\mathrm{D}\tau_i}{\mathrm{D}t} \right\rangle = \mathbf{\Lambda S}^{-1}\dot{\varepsilon}_i + \tau_i\omega_i - \omega_i\tau_i,$$

with $\dot{\varepsilon}_i = \frac{\langle \nabla u_i \rangle + \langle \nabla u_i \rangle^T}{2}$ and $\omega_i = \frac{\langle \nabla u_i \rangle - \langle \nabla u_i \rangle^T}{2}$.

Code

Source code for RootSPH model is hosted at the RootSPH repository

References

[1] Ted Belytschko et al. *Nonlinear finite elements for continua and structures*. John wiley & sons, 2013.

[2] MB Liu and GR Liu. "Smoothed particle hydrodynamics (SPH): an overview and recent developments". In: *Archives of computational methods in engineering* 17.1 (2010), pp. 25–76.

[3] Z.L. Zhang and M.B. Liu. "A decoupled finite particle method for modeling incompressible flows with free surfaces". In: *Applied Mathematical Modelling* 60 (2018), pp. 606–633. ISSN: 0307-904X. DOI: `https://doi.org/10.1016/j.apm.2018.03.043`. URL: `https://www.sciencedirect.com/science/article/pii/S0307904X18301677`.

Figure A. Anisotropy coefficient distribution for case 1.

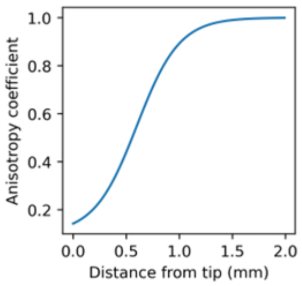

Figure B. Division threshold, softening coefficient, and anisotropy coefficient distributions for case 2 in relation to parameters x, s, and c.

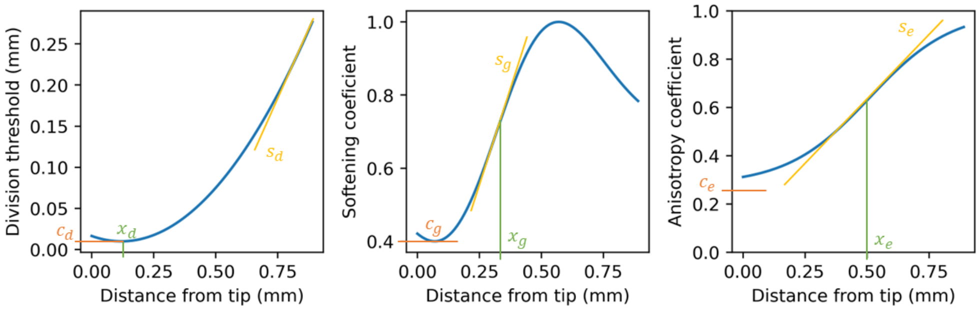

Supplement: S1 text — Method A. Detailed description of SPH theory. Figure A. Anisotropy coefficient distribution for the study of tissue anisotropy and the morphology of the root tip (Case 1). Figure B. Division threshold, softening coefficient, and anisotropy coefficient for the simulation of the development of Arabidopsis thaliana roots in unimpeded conditions (Case 2). (PDF) [file pcbi.1010916.s001.pdf]
